# Language experience influences performance on the NIH Toolbox Cognition Battery: A cluster analysis

**DOI:** 10.1057/s41599-025-04360-7

**Published:** 2025-01-18

**Authors:** Ashley Chung-Fat-Yim, Sayuri Hayakawa, Viorica Marian

**Affiliations:** 1 Northwestern University, Evanston, IL, USA.; 2 Oklahoma State University, Stillwater, OK, USA.

## Abstract

Studies investigating the effects of bilingualism on cognitive function have often yielded conflicting results, which may stem in part from the use of arbitrary criteria to categorize participants into groups based on language experience. The present study addresses this limitation by using a machine learning algorithm, known as cluster analysis, to identify naturally occurring subgroups of participants with similar language profiles. In a sample of 169 participants with varying degrees of first- and second-language proficiencies and ages of acquisition, the cluster analysis yielded four bilingual subgroups: late-unbalanced, early-unbalanced, late-balanced, and early-balanced. All participants completed the NIH Toolbox Cognition Battery. Results revealed that early-balanced and early-unbalanced bilinguals scored higher than late-unbalanced bilinguals on the cognitive flexibility and inhibitory control subtests of the NIH Toolbox Cognition Battery, whereas late-unbalanced bilinguals scored higher than early-balanced bilinguals on the verbal working memory subtest of the NIH Toolbox Cognition Battery. Bilingual language experience did not impact performance on measures of processing speed, episodic memory, and English vocabulary. These findings demonstrate the utility of data-driven approaches to capture the variability in language experience that exists in the real world. We conclude that different bilingual experiences can shape a wide range of cognitive abilities, from working memory to inhibitory control.

## Introduction

Bilingualism is on the rise with more than half of the world’s population using and speaking two or more languages on a regular basis (Marian, 2023). For example, in the United States, the number of dual-language programs has increased from 300 programs in 2001 to over 3600 programs in 2021 (Roberts, 2021). The language-learning app Duolingo reported over 500 million total users worldwide (Blanco, 2020). The impact of bilingualism on brain and cognitive development has been explored extensively (for reviews see Bialystok and Craik, 2022 and Marian et al. 2009). From better selective attention during infancy (Comishen et al. 2019; Kovács and Mehler, 2009) to delayed Alzheimer’s symptoms in older age (e.g., Alladi et al. 2013; Bialystok et al. 2007), the cognitive consequences associated with bilingualism can be profound. However, several studies have challenged these claims by showing equivalent performance between monolinguals and bilinguals on some cognitive tasks (e.g., Paap and Greenberg, 2013; von Bastian et al. 2016). Considering that many linguistic variables contribute to defining and measuring bilingualism, the current study used a data-driven approach to categorize participants into groups based on age of acquisition and proficiency in the first and second languages. These categorizations were used to explore how variations in bilingual experiences influence performance on a variety of cognitive tasks, including executive function (inhibition and cognitive flexibility), language (vocabulary and reading), memory (working memory and episodic memory), and processing speed.

New approaches to studying the relationship between bilingualism and cognition have emerged alongside an evolving understanding of bilingualism. To compare performance between monolinguals and bilinguals on cognitive tasks, mathematical approaches like ex-Gaussian distribution analysis (i.e., examining response time distributions, Calabria et al. 2011; Tse and Altarriba, 2014; Zhou and Krott, 2018), drift-diffusion modeling (i.e., decomposing response times into parameters of decision and non-decision times, Ong et al. 2017; Soares et al. 2019), and confirmatory factor analysis (Anjomshoae et al. 2024) have been used. To quantify the variability of language experience in bilingual populations, researchers have implemented statistical techniques on various language measures, such as computing a factor score (DeLuca et al. 2019), examining bilingualism as a continuous factor (Dash et al. 2019; Dash et al. 2022), or applying data reduction approaches (e.g., aggregation, principal components analyses, or language entropy; Gullifer and Titone, 2020; Gullifer et al. 2021; Kałamała et al. 2022).

The present study aimed to implement a statistical technique known as cluster analysis (MacQueen, 1967) to identify different types of linguistic profiles. Through an unsupervised learning algorithm, cluster analysis classifies individuals with shared characteristics or minimal variability in profiles and assigns them into groups based on these criteria. For example, a subgroup of participants, such as those who had been exposed to two languages from an early age, may perform better on the cognitive flexibility measure by virtue of greater experience managing multiple languages than a subgroup of participants who acquired a second language more recently. Likewise, speakers with greater asymmetry in proficiency between their first and second languages may perform better on the inhibitory control measure because they often need to inhibit the stronger and more dominant language. Theoretically, the findings from the cluster analysis may help establish a link between the underlying cognitive process and language control. Thus, cluster analysis provides a more nuanced analytical approach for categorizing participants than relying on arbitrary thresholds in proficiency level, age of acquisition, or usage.

## Consequences of Bilingualism for Language and Cognition

Previous research has shown that bilingualism has consequences for language processing (Beatty-Martínez and Dussias, 2017; Sulpizio et al. 2020). Some studies have reported that bilinguals tend to know fewer words in each language (e.g., Bialystok et al. 2022), are generally slower to name objects on picture naming tasks (e.g., Gollan et al. 2005; Ivanova and Costa, 2008; Poarch and van Hell, 2012), and generate fewer words on verbal fluency tasks (e.g., Bialystok et al. 2008; Friesen et al. 2015; Sandoval et al. 2010) than their monolingual counterparts. Because bilinguals are dividing their time between multiple languages, the bilingual’s weaker performance on verbal tasks may be attributed to their less frequent use of and exposure to each language compared to speakers of a single language. Additionally, bilinguals co-activate the lexicons from both of their languages even when only one language is needed (Marian and Spivey, 2003; Shook and Marian, 2019; Van Heuven et al. 1998; Wu and Thierry, 2010). Hence, the bilingual language system must manage competition between languages by selecting words in the intended language while suppressing or inhibiting those in the irrelevant language (Green, 1998). The amount of inhibition required to suppress the irrelevant language depends on the level of proficiency in each language. For bilinguals with a strong first language and weaker second language, the amount of inhibition needed to suppress the first language when operating in the second language will be larger than the amount of inhibition needed to suppress the second language when operating in the first language (Meuter and Allport, 1999).

Although the co-activation of competing languages may contribute to greater difficulty in some contexts (e.g., for word retrieval), the same mechanism has been proposed to underlie several reported benefits for cognitive function. Because bilinguals have two languages at their disposal for every communicative interaction, greater demands are placed on the fronto-parietal network to manage attention across two simultaneously active language systems. The predominant view is that language control in bilinguals is carried out by the executive control network (e.g., Anderson et al. 2018; Chen et al. 2021; De Baene et al. 2015). The accrued practice using executive control processes to ignore the irrelevant language while selecting the relevant language may enhance the domain-general executive control network for purposes beyond language.

Miyake and colleagues (2000) proposed a model of executive function consisting of three components: inhibiting prepotent responses, shifting between tasks, and updating working memory representations. On nonverbal executive control tasks of inhibition and shifting, bilinguals are generally faster and more accurate than monolinguals (see Bialystok, 2017 for a review). A wide range of linguistic factors, including the age of second language acquisition (Kapa and Colombo, 2013; Luk et al. 2011), second language proficiency (Rosselli et al. 2016; Tse and Altarriba, 2014; Videsott et al. 2012), and language control abilities (i.e., switchers versus non-switchers; Festman et al. 2010) have been shown to modulate executive control. In adults, language group effects are less pronounced on executive control tasks because accuracy tends to be at ceiling (Bialystok, 2017). Due to the lack of variability in performance and because both groups are operating at peak efficiency, bilingualism may not be associated with better performance on nonverbal measures of executive control in young adults.

Several meta-analyses have revealed that bilingualism was associated with better working memory abilities with a moderate effect size (Adesope et al. 2010; Grundy and Timmer, 2017; Monnier et al. 2022). Specifically, bilinguals generally achieve higher levels of performance than monolinguals when the working memory task is *nonverbal* (e.g., Cockroft et al. 2019; Hansen et al. 2016; Luo et al. 2013), effortful (Antón et al. 2019; Jiao et al. 2019; Morales et al. 2013), and performed in the bilingual’s native language (Grundy and Timmer, 2017). However, not all studies find that bilingualism leads to cognitive gains in working memory. Equivalent performance between language groups has been observed in children (McVeigh et al. 2019) and adults (D’Souza et al. 2018; Ratiu and Azuma, 2015) on working memory tasks.

Juggling multiple languages on a regular basis may enhance domain-general executive control, cascading into general improvements in processing speed, which has been described as the ability to identify, process, and formulate a response to information in a set amount of time (Kail and Salthouse, 1994). Donnelly and colleagues (2019) conducted a meta-analysis of 80 studies and found a small significant effect in favor of bilinguals for global response times (RTs) on nonverbal executive control tasks (e.g., Simon, Flanker, and Stroop tasks), with faster RTs for bilinguals than monolinguals on *both* congruent and incongruent trials. Furthermore, Luque and Morgan‐Short (2021) found an association between degree of second-language proficiency in adult learners of Spanish and processing speed on the AX-CPT task, with greater proficiency in a second language associated with faster responses. Although some studies have shown that fluency in multiple languages influences how fast information is processed on executive control tasks, only a small number of studies have either included a pure measure of processing speed in the testing battery (Bonifacci et al. 2011; Ebert, 2021), accounted for processing speed in the analyses (Blumenfeld et al. 2016), or matched monolingual and bilingual participants on processing speed (Marton, 2016). Considering that the effect of bilingualism on pure measures of processing speed remains largely unknown, the current study examined how language experience affects processing speed.

Preliminary evidence suggests that bilingualism can additionally impact episodic memory. Episodic memory refers to the recollection of personal experiences, including details about the event’s time and location. For bilinguals, episodic memory may include information about the linguistic context (Fernandez-Duque et al. in press; Schroeder and Marian, 2014). Bilinguals are more accurate at retrieving memories when the language at encoding is the same as the language at retrieval (Marian and Kaushanskaya, 2007). To date, only a few studies have directly compared bilinguals to monolinguals on episodic memory tasks (but see Ljungberg et al. 2013, 2020; Schroeder and Marian, 2012). For example, Schroeder and Marian (2012) found that bilinguals outperformed monolinguals on an episodic memory task, with earlier ages of second language acquisition and greater exposure to the second language leading to higher recall accuracy. These findings suggest that bilingualism can shape episodic memory.

## NIH Toolbox Cognition Battery

The NIH Toolbox Cognition Battery (Zelazo et al. 2013) assesses a wide range of cognitive abilities, including language, executive function, memory, and processing speed, in individuals from ages 3 to 85. The effects of bilingualism on performance in the NIH Toolbox Cognition Battery have been explored in various age populations, including children (Haft et al. 2019; Kuzyk et al. 2020; Nayak and Tarullo, 2020), adolescents (Dick et al. 2019; Kwon et al. 2021; Vaughn et al. 2021), and young adults (Marian et al. 2018a). In the young population, the differences between language groups on the individual subtests of the NIH Toolbox Cognition Battery appear to be small. Marian and colleagues (2018a) observed that bilinguals had higher scores on the Flanker inhibitory control test, but lower scores on the List Sorting working memory test than monolinguals. Although unexpected given previous work showing a positive impact of bilingualism on working memory (e.g., Grundy and Timmer, 2017; Monnier et al. 2022), the lower scores on the List Sorting working memory test for bilinguals may have been due to their lower proficiency in English. Considering that the list sorting test involves recalling items in English, the verbal working memory task may be tapping into English comprehension or vocabulary knowledge rather than working memory.

## The Present Study

The present study uses cluster analysis to identify individuals with similar linguistic profiles. Clustering is an unsupervised machine learning algorithm for identifying subgroups of observations within a dataset (MacQueen, 1967). After mapping each participant within an *n*-dimensional coordinate space (e.g., by proficiency on the y-axis and age of acquisition on the x-axis), the algorithm begins by randomly selecting *k* centroid values from the dataset (corresponding to the specified number of clusters) and calculating the Euclidean distance between each remaining point and the closest centroid. The centroid is then reassigned as the mean of the cluster and the distance between each point and centroid is recalculated. Through an iterative process, the clusters and centroids are adjusted to minimize the distance between points within a given cluster and maximize the distance between points in different clusters. Ultimately, the goal is to identify subgroups of participants with similar language profiles to each other. This method has been implemented in almost every field from vision (e.g., Dhanachandra et al. 2015) to business (e.g., Anitha and Patil, 2022; Wang et al. 2005) to medicine (e.g., Juang and Wu, 2010; Khanmohammadi et al. 2017; Wismüller et al. 2002). In bilingualism research, cluster analysis has been used to identify language impairments in bilingual children (Hamann and Ibrahim, 2017) and to determine whether cognitive profiles can classify individuals as monolinguals and bilinguals (Jones et al. 2021). The results from the cluster analysis will uncover the types of cognitive resources recruited by bilinguals with similar linguistic profiles.

After identifying subgroups of participants, we will compare their performance on each subtest of the NIH Toolbox Cognition Battery that measure executive function (inhibitory control and cognitive flexibility), language (English reading and vocabulary), memory (episodic memory and working memory), and processing speed. For the cluster analysis, it is hypothesized that four clusters will emerge: early-unbalanced, early-balanced, late-balanced, and late-unbalanced bilinguals. Previous research has shown that bilinguals tend to perform worse than monolinguals on verbal measures (Bialystok et al. 2022; Gollan et al. 2005; Ivanova and Costa, 2008), but better on nonverbal executive control (e.g., inhibitory control and cognitive flexibility; Bialystok, 2017; Marian et al. 2009) and memory tasks (e.g., working memory and episodic memory; Grundy and Timmer, 2017; Monnier et al. 2022; Schroeder and Marian, 2012). Based on these findings, it is hypothesized that early-balanced bilinguals will perform worse than late-unbalanced bilinguals and monolinguals on verbal measures, but perform better than both groups on nonverbal executive control and memory tasks. As proficiency increases in both languages (i.e., balanced bilingualism), the bilingual individual may need to recruit executive control to prevent intrusions from the nontarget language. Similarly, managing multiple languages from an early age (i.e., early bilingualism) allows for extensive practice in attending to and selecting the target language. We predicted that early bilingualism and more balanced proficiency would result in better performance on nonverbal tasks of executive control, but worse performance on verbal tasks of executive control.

An open question, however, is the degree of interactivity between proficiency and age of acquisition. Although both variables were predicted to impact verbal and nonverbal cognitive function, few studies have examined the independent and interacting effects of earlier second language acquisition and more balanced language proficiency on cognitive performance within the same study and in young adult populations (Yow and Li, 2015). Thus, the examination of early-unbalanced and late-balanced bilinguals in addition to early-balanced and late-unbalanced bilinguals will make it possible to investigate whether proficiency and age of acquisition have additive or compensatory effects on cognitive function. For instance, if the *strength* of language co-activation and competition in daily life (which should increase with higher bilingual proficiency) and the *duration* of bilingual experience (i.e., age of bilingual acquisition) have independent positive effects on nonverbal executive control, we may expect an additive effect whereby early-balanced bilinguals outperform both early-unbalanced and late-balanced bilinguals. On the other hand, if the strength and duration of experience managing two languages each serve to strengthen nonverbal executive control via the same mechanism, we may expect a compensatory effect whereby either form of experience is sufficient to elicit improved performance (i.e., equivalent performance among early-balanced, early-unbalanced, and late-balanced bilinguals).

## Method

### Participants.

Data from 169 participants were compiled across four existing datasets from our lab (Chabal et al. 2022; Chen et al. 2017; Hayakawa et al. 2020; Marian et al. 2018b). These datasets were selected because they included data from the Language Experience and Proficiency Questionnaire (LEAP-Q; Marian et al. 2007) and individual test scores from the NIH Toolbox Cognition Battery (Zelazo et al. 2013). The LEAP-Q was used to obtain information regarding each participant’s language usage patterns as well as their level of proficiency, age of acquisition, and degree of exposure in each language. Participants rated their level of proficiency in each language on a scale from 0 (None) to 10 (Perfect) in speaking, understanding, and reading. They rated the extent to which several factors contributed towards learning each language (i.e., manner of acquisition) on a scale from 0 (Not a Contributor) to 10 (Most Important Contributor). The factors included interacting with friends, interacting with family, watching TV, reading, language tapes/self-instruction, and listening to the radio. Additionally, participants rated the extent to which they are exposed to each language (i.e., current exposure) on a scale from 0 (Never) to 10 (Always) in different contexts, including friends, family, TV, radio/music, reading, and language-lab/self-instruction. For each language, participants reported the number of years and months spent in a country, family, and school and/or work environment where the language is spoken (i.e., immersion).

Monolinguals were English native speakers with minimal proficiency in a second language (mean ratings of 0 or 1 out of 10 in second language proficiency averaged across speaking, understanding, and reading; *N* = 29). The remaining 140 participants with self-reported second language proficiency of 2 or higher were proficient in English and had experience with one of the following languages: Spanish (*N* = 63), Korean (*N* = 51), French (*N* = 10), Chinese (*N* = 4), American Sign Language, German, Hebrew, Italian (*N* = 2 each), Ikwerre, Polish, Russian, and Tagalog (*N* = 1 each). Out of 140 bilinguals, English was the L1 (first language acquired) for 83 bilinguals and English was the L2 (second language acquired) for 57 bilinguals. On average, bilinguals rated their proficiency in their L1 as 9.35 (*SD* = 0.97) and L2 as 6.28 (*SD* = 2.90) out of 10. Monolinguals significantly differed from bilinguals on all L2 measures other than the duration immersion in an L2-speaking family. Monolinguals and bilinguals significantly differed on all L1 measures of proficiency, age of acquisition, current exposure, and immersion (*p*s < 0.05). Sixty-one participants reported knowing three or more languages. All participants were adults between the ages of 18 and 36. Monolinguals and bilinguals did not differ in gender (*Ms* = 81.5% and 72.1% female, respectively; *p* = 0.32), age in years (*Ms* = 22.64 and 21.56, respectively; *p* = 0.13), or years of education (*Ms* = 15.79 and 15.07, respectively; *p* = 0.10). Language background information are reported for each language group in Table 1.

Participants provided consent and were debriefed about the objective of the study at the time of testing. The individual studies included in these analyses received ethics approval from Northwestern University’s Institutional Review Board. Across studies, participants were recruited through university-wide mailing lists, flyers around campus, or the undergraduate research participant pool. Participants either received course credit or monetary compensation for their time.

### NIH Toolbox Cognition Battery (Zelazo et al. 2013).

The NIH Toolbox Cognition Battery is a standardized assessment tool used to measure a variety of different cognitive processes, including language, executive function, memory, and processing speed. The individual tests were administered and scored according to the guidelines in the testing manual. Raw scores were converted to normalized scaled scores by age (Casaletto et al. 2015). The battery took approximately 30 to 35 min to complete.

Reliability and validity were previously assessed by Weintraub et al. (2013). The authors ran interclass correlations between each test and well-established measures of the same construct (e.g., Picture Vocabulary Test from the NIH Toolbox Cognition Battery and the Peabody Picture Vocabulary Test – Fourth Edition by Dunn and Dunn, 2007). In adults, the interclass correlations were high, ranging from 0.72 for processing speed to 0.94 for inhibitory control. Furthermore, correlations for convergent validity were high, suggesting that each measure taps into the desired construct, while correlations for discriminant validity were low, indicating a weak relationship with measures tapping into different constructs (Weintraub et al. 2013).

#### Executive Function.

Inhibitory control was measured using the Flanker task (Eriksen and Eriksen, 1974). In the adult version of the Flanker task, participants were shown a row of five arrows. The middle arrow pointed either in the same direction (←←←←←) or opposite direction (←←→←←) as the surrounding arrows. Participants selected the button that matched the direction the middle arrow was pointing to and completed four practice trials with feedback and 20 experimental trials in approximately 4 min.

Cognitive flexibility was measured using the Dimensional Change Card Sort test (DCCS; Zelazo, 2006). In the adult version of the DCCS test, participants sorted bivalent stimuli (e.g., blue ball or yellow truck) by either its shape or colour depending on the cue provided. Four practice trials and 30 experimental trials with color and shape cues intermixed were administered to participants. On average, the test took approximately 4 min to complete.

#### Language.

Receptive vocabulary in English was measured using the Picture Vocabulary test and decoding skills in reading was measured using the Oral Reading Recognition test. In the Picture Vocabulary test, participants selected an image out of four options that best represented the word they heard. Two practice trials and approximately 25 items were administered to participants. On average, the test took 4 min to complete.

For the Oral Reading Recognition test, participants had 120 seconds to name out loud a series of letters or words that were presented on the screen one at a time. The number of items shown was based on the age of the participant, but on average, around 30 to 40 items were presented to participants.

#### Memory.

Working memory was assessed using the List Sorting Working Memory test and episodic memory was assessed using the Picture Sequence Memory test. The List Sorting Working Memory test consisted of two conditions: 1-List condition and 2-List condition. In the 1-List condition, participants were shown pictures one at a time that belonged to the same category (e.g., fruits) followed by a blank screen. Once the blank screen appeared, participants named the pictures from smallest to largest. The 2-List condition followed a similar procedure as the 1-List condition, except that the pictures were from two different categories (e.g., animals and fruits). When the blank screen appeared, participants named the pictures from smallest to largest from the first category (e.g., animals) followed by the second category (e.g., fruits). Participants were administered two practice trials with two pictures in each series. For the experimental trials, the number of pictures within each series increased by one on trials following a correct response (from two pictures to a maximum of seven pictures in a single sequence). The test ended when 2 trials of the same length failed or the maximum sequence of seven items was presented. On average, the test took approximately 7 min to complete.

In the Picture Sequence Memory test (episodic memory), participants were shown a sequence of pictures containing specific events or activities, such as going camping, having a birthday party, playing in the park, and so on. Once the sequence ended, the pictures were scrambled, and participants were instructed to move the pictures to the location they saw them move to on the screen. Participants were provided with one practice sequence, containing four pictures. If the participant succeeded in four trials, two experimental sequences were presented: one with 15 pictures and the other with 18 pictures. They completed the test in approximately 10 min.

#### Processing Speed.

The Pattern Comparison Processing Speed test was used to measure processing speed. Two pictures appeared on the screen side-by-side. If the two pictures were identical, participants touched the “Yes” button. If the two pictures were different, participants touched the “No” button. Participants were administered six practice trials and named as many items as possible (of a possible 130) within 85 seconds.

### Statistical Analyses

#### Cluster Analysis.

A cluster analysis was conducted to determine the optimal grouping among the 140 bilingual participants who reported proficiency greater than 1 (out of 10) in a second language. As information regarding manner of acquisition, exposure, and/or immersion were missing from 54 of the 140 bilingual participants, clusters were determined based on mean-centered measures of relative age of acquisition (L2 – L1) and relative proficiency (L1 – L2). Analyses were carried out using the Hartigan-Wong K-means clustering algorithm (Hartigan and Wong, 1979), executed with the *kmeans* R function with 20 random starting assignments and 4 *k* clusters (based on an examination of within-cluster sum of squares to determine the optimal number of groups).

To obtain more information about each group’s language usage patterns, we also examined whether the groups differed in their manner of language acquisition and exposure. Following an examination of correlations among LEAP-Q measures, four composite measures were created capturing 1) family acquisition and exposure (aggregated across manner of acquisition and current exposure ratings for family), 2) media and community language acquisition (aggregated across manner of acquisition ratings for friends, reading, TV, and music), 3) media and community language exposure (aggregated across current exposure ratings for friends, reading, TV, and music), and 4) language immersion (aggregated across the number of years immersed in a country, school, and work environment in which a given language was spoken) for each language.

#### NIH Toolbox Analyses.

Separate linear models examined the effect of cluster group (monolinguals, late-unbalanced bilinguals, early-unbalanced bilinguals, late-balanced bilinguals, early-balanced bilinguals; see Results for additional detail) on each of the NIH Toolbox Cognition Battery measures. For each model, cluster group was sum coded with four contrasts (monolingual: 1, 0, 0, 0; late-unbalanced bilinguals: 0, 1, 0, 0; early-unbalanced bilinguals: 0, 0, 1, 0; late-balanced bilinguals: 0, 0, 0, 1; early-balanced bilinguals: −1, −1, −1, −1) to compare each group to the grand mean. Each group was compared to the grand mean because there was no group that could theoretically serve as the reference level. Following initial comparisons to the grand mean, we performed Tukey-adjusted planned pairwise comparisons to compare each group to each other using the *emmeans* R function (Lenth et al. 2018).

The bilingual clusters did not differ in gender (*ps* > 0.12) or in age (*p*s > 0.090). However, because monolinguals (*M* = 22.64, *SD* = 8.46) were older than late-unbalanced bilinguals (*M* = 21.00, *SD* = 2.45, *p* = 0.036) and marginally older than early-balanced bilinguals (*M* = 21.22, *SD* = 3.15, *p* = 0.069), all models included age as a covariate. Furthermore, as late-unbalanced bilinguals (*M* = 14.59, *SD* = 2.65) had fewer years of education relative to the grand mean (*M* = 15.19, *SD* = 2.11; *Estimate* = −0.65, *SE* = 0.32, *t*(163) = −2.02, *p* = 0.045), all models included mean-centered years of education as a covariate. Years of education did not significantly differ among monolinguals (*M* = 15.79, *SD* = 2.08), early-unbalanced bilinguals (*M* = 15.22, *SD* = 2.00), late-balanced bilinguals (*M* = 15.60, *SD* = 1.83), or early-balanced bilinguals (*M* = 15.02, *SD* = 1.85), *ps* > 0.12. All unbalanced bilinguals had English as their L1, whereas the balanced bilinguals had a mixture of English as their L1 or the other language as their L1. To account for the potential differences between balanced and unbalanced bilinguals in English proficiency, we included average English proficiency (aggregated across English speaking, understanding, and reading proficiency) as a covariate in all models. Moreover, as 61 of the participants reported knowing three or more languages, we also included number of known languages as a covariate in all models.

## Results

**Fig. 1:**
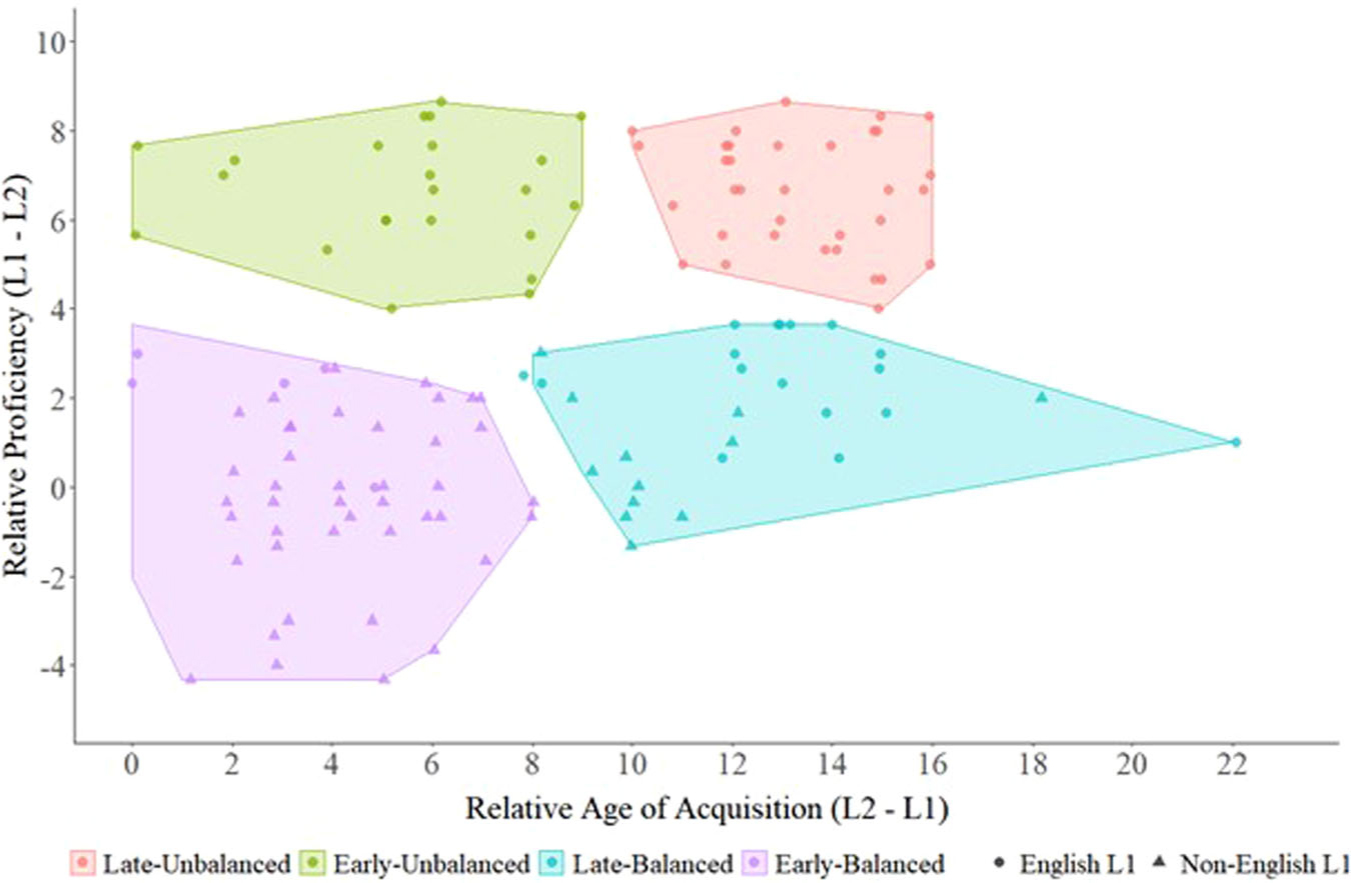
Cluster analyses based on relative age of acquisition (L2 – L1) and proficiency (L1 – L2) yielded four bilingual groups comprised of late-unbalanced bilinguals (in red; *N* = 34), early-unbalanced bilinguals (in green; *N* = 23), late-balanced bilinguals (in teal; *N* = 29), and early-balanced bilinguals (in purple; *N* = 54). Circles represent participants whose L1 was English and triangles represent participants whose L1 was a non-English language.

The cluster analysis based on relative language proficiency (L1 – L2) and relative age of acquisition (L2 – L1) yielded four bilingual groups, which broadly corresponded to late-unbalanced bilinguals (*N* = 34), early-unbalanced bilinguals (*N* = 23), late-balanced bilinguals (*N* = 29), and early-balanced bilinguals (*N* = 54; see Fig. 1 as well as Tables 2 and 3 for overviews of each group’s linguistic profile). The total number of participants contributing to each model varied depending on the availability of relevant NIH Toolbox Cognition Battery measures.

The difference between L1 and L2 age of acquisition (AoA) was significantly smaller for early (aggregated across balanced and unbalanced bilinguals; *M* = 4.21, *SD* = 2.53) compared to late bilinguals (*M* = 12.84, *SD* = 2.5; *Estimate* = −8.63, *SE* = 0.7, *t*(166) = −12.29, *p* < 0.001). The difference between L1 and L2 proficiency was significantly smaller for balanced bilinguals (aggregated across early and late bilinguals; *M* = 0.63, *SD* = 2.02) compared to unbalanced bilinguals (*M* = 6.63, *SD* = 1.28; *Estimate* = 6.00, *SE* = 0.28, *t*(166) = 21.43, *p* < 0.001). Although L1 and L2 AoA were closer for early than late bilinguals, L1 AoA was significantly earlier than L2 AoA for all four groups (*ps* < 0.001). L1 proficiency was significantly higher than L2 proficiency for early-unbalanced, late-unbalanced, and late-balanced bilinguals (*ps* < 0.001), whereas L1 and L2 proficiency did not differ for early-balanced bilinguals (*p* = 0.89). English was the L1 for all late-unbalanced and early-unbalanced bilinguals. English was the L1 for 58.6% of late-balanced bilinguals and 16.7% of early-balanced bilinguals. English AoA was significantly earlier than non-English AoA for early- and late-unbalanced bilinguals (*ps* < 0.001), did not differ for late-balanced bilinguals (*p* = 0.16), and was significantly later than non-English AoA for early-balanced bilinguals (*p* < 0.001). English proficiency was significantly greater than non-English proficiency for all four groups (*ps* < 0.007).

### Executive Function

#### Flanker Task: Inhibitory Control.

**Fig. 2:**
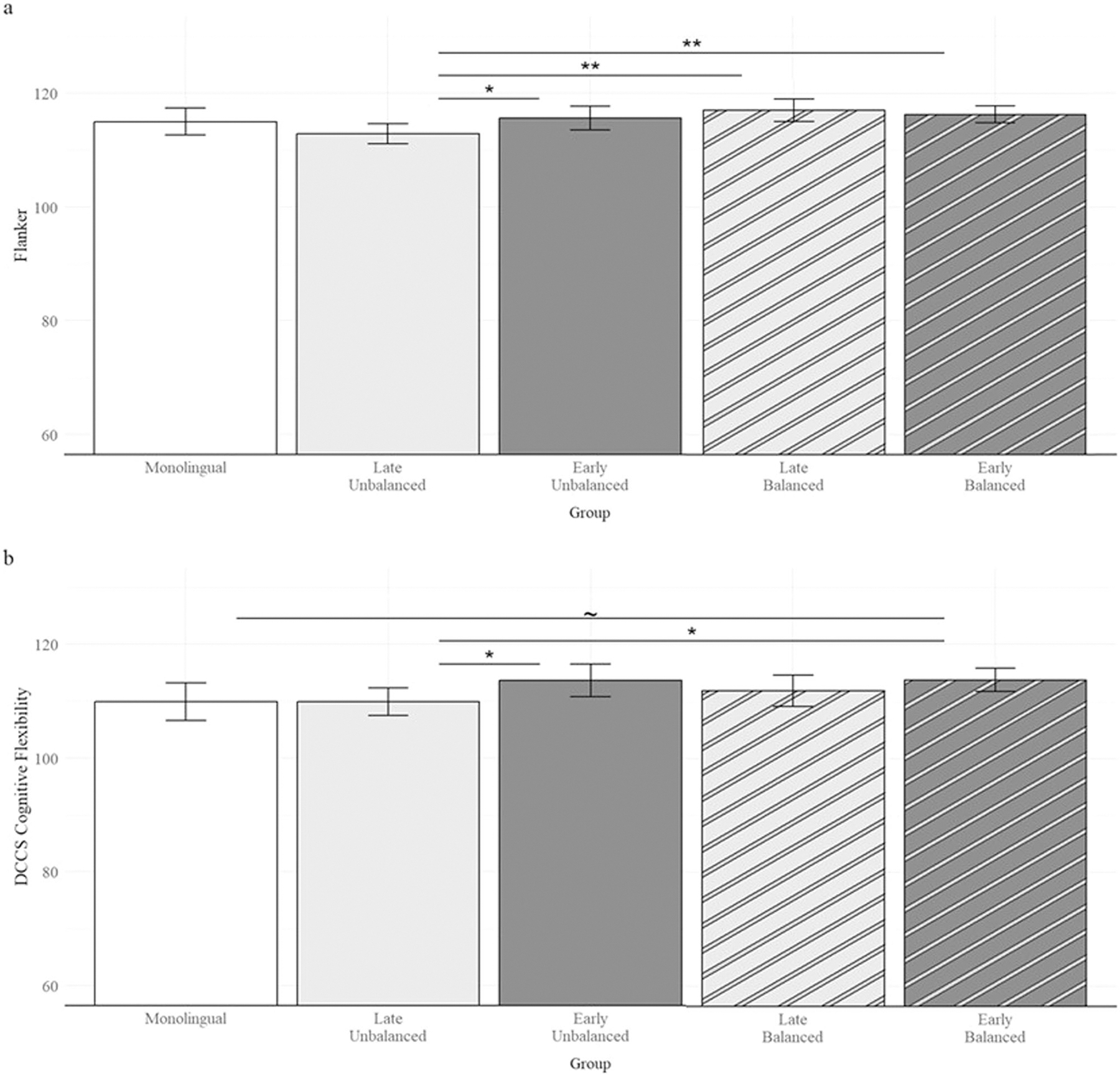
Mean scores on the executive function subtests of the NIH Toolbox Cognition Battery for each language group. Flanker scores (**a**) were higher among early-balanced, late-balanced, and early-unbalanced bilinguals relative to late-unbalanced bilinguals. DCCS scores (**b**) were higher among early-unbalanced and early-balanced bilinguals relative to late-unbalanced bilinguals, and marginally higher among early-balanced bilinguals relative to monolinguals. Error bars represent 95% confidence intervals. **p < 0.01, *p < 0.05, ~*p* < 0.07.

A total of 156 participants contributed to the model for flanker performance, including 22 monolinguals, 34 late-unbalanced bilinguals, 23 early-unbalanced bilinguals, 25 late-balanced bilinguals, and 52 early-balanced bilinguals. An additional 13 participants were not included in the analysis due to missing scores (*n* = 6) or due to scores more than 2 standard deviations below the grand mean (*n* = 7). Participants had a mean Flanker score of 115.37 (*SD* = 5.31) and late-unbalanced bilinguals had significantly lower scores compared to the grand mean (*Estimate* = 2.50, *SE* = 0.81, *t*(147) = 3.11, *p* = 0.0023). Tukey-adjusted planned pairwise comparisons revealed that late-unbalanced bilinguals had significantly lower scores than early-unbalanced bilinguals (*Estimate* = 2.75, *SE* = 1.37, *t*(147) = 2.02, *p* = 0.046, *Cohen*’*s d* = 0.55, 95% CI [−1.09, −0.01]), late-balanced (*Estimate* = 4.20, *SE* = 1.35, *t*(147) = 3.11, *p* = 0.002, *Cohen*’*s d* = 0.84, 95% CI [−1.38, −0.30]), and early-balanced bilinguals (*Estimate* = 3.45, *SE* = 1.17, *t*(147) = 2.95, *p* = 0.004, *Cohen*’*s d* = 0.69, 95% CI [−1.15, −0.22]; see Fig. 2a). Late-balanced, early-balanced, and early-unbalanced bilinguals were not significantly different from each other, *p*s > 0.33. Furthermore, monolinguals did not differ from any of the bilingual groups, all *p*s > 0.15. Flanker scores declined with age (*Estimate* = 0.38, *SE* = 0.17, *t*(147) = 2.21, *p* = 0.028), but not with years of education, number of known languages, or average English proficiency, all *ps* > 0.25.

#### Dimensional Change Card Sort (DCCS) Test: Cognitive Flexibility.

A total of 151 participants contributed to the model for DCCS performance, including 20 monolinguals, 33 late-unbalanced bilinguals, 23 early-unbalanced bilinguals, 24 late-balanced bilinguals, and 51 early-balanced bilinguals. An additional 18 participants were not included in the analysis due to missing scores (*n* = 9) or due to scores greater than 2 standard deviations below the grand mean (*n* = 9). Participants had a mean DCCS score of 112.09 (*SD* = 6.92). None of the groups’ scores were significantly different from the grand mean, *p*s > 0.085. Tukey-adjusted planned pairwise comparisons revealed early-unbalanced bilinguals (*Estimate* = 3.75, *SE* = 1.86, *t*(142) = 2.01, *p* = 0.046, *Cohen*’*s d* = 0.55, 95% CI [−1.10, −0.01]) and early-balanced bilinguals (*Estimate* = 3.76, *SE* = 1.60, *t*(142) = 2.34, *p* = 0.020, *Cohen*’*s d* = 0.57, 95% CI [−1.04, −0.10]) scored significantly higher than late-unbalanced bilinguals (Fig. 2b). The other bilingual groups did not differ significantly from each other, *p*s > 0.27. Additionally, monolinguals scored marginally lower than early-balanced bilinguals (*Estimate* = 3.88, *SE* = 2.08, *t*(142) = 1.86, *p* = 0.064, *Cohen*’*s d* = 0.57, 95% CI [−1.18, 0.038]), but did not significantly differ from any of the other bilingual groups, *p*s > 0.091. DCCS scores did not vary by age, years of education, or number of known languages (*ps* > 0.29), but did vary marginally by average English proficiency (*Estimate* = 1.40, *SE* = 0.75, *t*(142) = 1.85, *p* = 0.066).

### Language

#### Picture Vocabulary.

A total of 162 participants contributed to the model for English picture vocabulary performance, including 24 monolinguals, 34 late-unbalanced bilinguals, 22 early-unbalanced bilinguals, 29 late-balanced bilinguals, and 53 early-balanced bilinguals. An additional 7 participants were not included in the analysis due to missing scores (*n* = 4) or due to scores greater than 2 standard deviations below the grand mean (*n* = 3). Participants had a mean Picture Vocabulary score of 116.04 (*SD* = 12.80). None of the groups’ scores were significantly different from the grand mean, *p*s > 0.17. Tukey-adjusted planned pairwise comparisons revealed that scores did not significantly differ among monolinguals (*M* = 116.69, *SD* = 14.05), late-unbalanced bilinguals (*M* = 118.96, *SD* = 10.74), early-unbalanced bilinguals (*M* = 116.99, *SD* = 10.50), late-balanced bilinguals (*M* = 116.78, *SD* = 11.57), and early-balanced bilinguals (*M* = 113.07, *SD* = 14.65), *p*s > 0.087. Picture Vocabulary scores did not vary with age, years of education, number of known languages, or average English proficiency, *ps* > 0.14.

#### Oral Reading Recognition.

A total of 95 participants contributed to the model for English oral reading performance, including 11 monolinguals, 28 late-unbalanced bilinguals, 16 early-unbalanced bilinguals, 13 late-balanced bilinguals, and 27 early-balanced bilinguals. An additional 74 participants were not included in the analysis due to missing scores (*n* = 68) or due to scores greater than 2 standard deviations below the grand mean (*n* = 6). Participants had a mean Oral Reading score of 120.82 (*SD* = 8.15) and late-unbalanced bilinguals (*M* = 123.54, *SD* = 6.97) had marginally higher scores than the grand mean (*Estimate* = −2.97, *SE* = 1.5, *t*(86) = 1.98, *p* = 0.051). Tukey-adjusted planned pairwise comparisons revealed that oral reading performance was marginally higher for late-unbalanced bilinguals relative to late-balanced bilinguals (*M* = 118.22, *SD* = 10.60; *Estimate* = −4.90, *SE* = 2.76, *t*(86) = −1.78, *p* = 0.079, *Cohen*’*s d* = 0.60, 95% CI [−0.076, 1.28]). Early-unbalanced (*M* = 120.41, *SD* = 7.80) and early-balanced (*M* = 120.07, *SD* = 8.31) bilinguals did not differ significantly from any of the bilingual groups, *p*s > 0.22. The monolinguals (*M* = 119.39, *SD* = 7.34) did not differ from any of the bilingual groups, *p*s > 0.14. Oral Reading Recognition scores did not vary with age, years of education, number of known languages, or average English proficiency, *ps* > 0.25.

### Memory

#### List Sorting Working Memory.

**Fig. 3:**
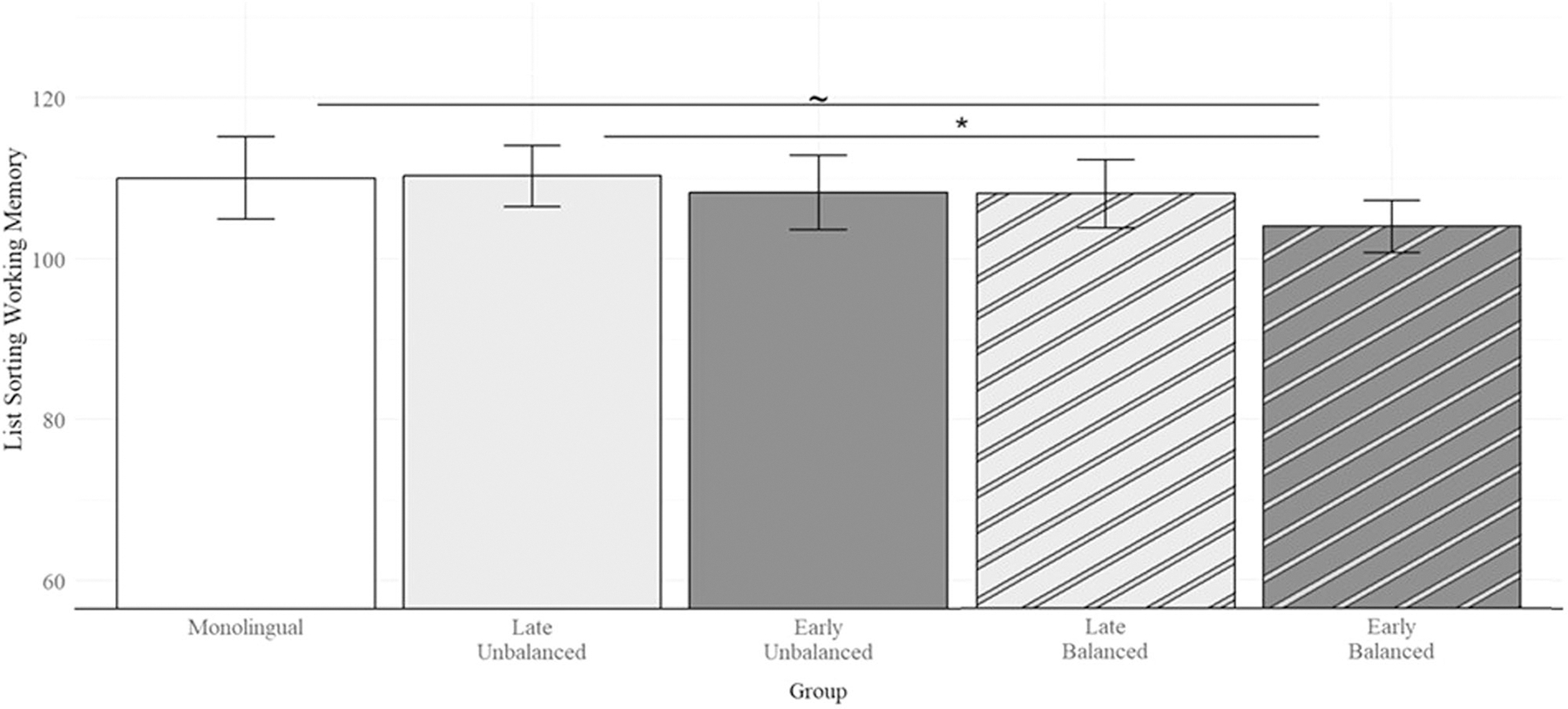
Mean List Sorting Working Memory scores by group. List sorting scores were higher among late-unbalanced bilinguals relative to early-balanced bilinguals, and marginally higher among monolinguals relative to early-balanced bilinguals. Error bars represent 95% confidence intervals. **p < 0.01, *p < 0.05, ~*p* < 0.07.

A total of 159 participants contributed to the model for list sorting working memory performance, including 23 monolinguals, 34 late-unbalanced bilinguals, 23 early-unbalanced bilinguals, 28 late-balanced bilinguals, and 51 early-balanced bilinguals. An additional 10 participants were not included in the analysis due to missing scores (*n* = 8) or due to scores greater than 2 standard deviations below the grand mean (*n* = 2). Participants had a mean List Sorting Working Memory score of 107.55 (*SD* = 11.45) and early-balanced bilinguals had significantly lower scores than the grand mean (*Estimate* = −4.11, *SE* = 1.65, *t*(150) = −2.50, *p* = 0.014). Tukey-adjusted planned pairwise comparisons revealed that early-balanced bilinguals scored significantly lower than late-unbalanced bilinguals (*Estimate* = −6.26, *SE* = 2.56, *t*(150) = −2.44, *p* = 0.016, *Cohen*’*s d* = 0.56, 95% CI [0.10, 1.03]) and marginally lower than monolinguals (*Estimate* = −6.02, *SE* = 3.22, *t*(150) = −1.87, *p* = 0.064, *Cohen*’*s d* = 0.54, 95% CI [−0.034, 1.12], see Fig. 3). All other bilingual groups did not differ from each other, *p*s > 0.13. Additionally, monolinguals did not differ from late-balanced, late-unbalanced, and early-unbalanced bilinguals, *p*s > 0.57. List Sorting Working Memory scores did not decline with age, years of education, number of known languages, or average English proficiency, *p*s > 0.14.

#### Picture Sequence Episodic Memory.

A total of 100 participants contributed to the categorical model for picture sequence episodic memory performance, including 10 monolinguals, 28 late-unbalanced bilinguals, 16 early-unbalanced bilinguals, 15 late-balanced bilinguals, and 31 early-balanced bilinguals. An additional 69 participants were not included in the analysis due to missing scores (*n* = 65) or due to scores greater than 2 standard deviations below the grand mean (*n* = 4). Participants had a mean picture sequence episodic memory score of 112.82 (*SD* = 13.68). Tukey-adjusted planned pairwise comparisons revealed that scores did not significantly differ among monolinguals (*M* = 113.50, *SD* = 14.46), late-unbalanced bilinguals (*M* = 111.69, *SD* = 15.06), early-unbalanced bilinguals (*M* = 114.39, *SD* = 13.57), late-balanced bilinguals (*M* = 115.48, *SD* = 12.66), and early-balanced bilinguals (*M* = 111.52, *SD* = 13.26), *ps* > 0.32. Picture Sequence Episodic Memory scores did not decline with age, years of education, number of known languages, or average English proficiency, *ps* = 0.20.

### Pattern Comparison Processing Speed.

A total of 153 participants contributed to the model for pattern comparison processing speed performance, including 22 monolinguals, 33 late-unbalanced bilinguals, 22 early-unbalanced bilinguals, 27 late-balanced bilinguals, and 49 early-balanced bilinguals. An additional 16 participants were not included in the analysis due to missing (*n* = 8) scores or due to scores greater than 2 standard deviations below the grand mean (*n* = 8). Participants had a mean Pattern Comparison score of 126.22 (*SD* = 16.55). Tukey-adjusted planned pairwise comparisons revealed that scores did not significantly differ among monolinguals (*M* = 125.86, *SD* = 16.25), late-unbalanced bilinguals (*M* = 123.39, *SD* = 19.05), early-unbalanced bilinguals (*M* = 122.41, *SD* = 13.50), late-balanced bilinguals (*M* = 128.02, *SD* = 17.31), and early-balanced bilinguals (*M* = 129.02, *SD* = 15.67), *p*s > 0.11. Patten Comparison Processing Speed scores declined with age (*Estimate* = −1.37, *SE* = 0.56, *t*(144) = −2.45, *p* = 0.015), but not years of education, number of known languages, or average English proficiency, *p*s > 0.70.

## Discussion

The goal of the present study was to investigate the impact of language experience on cognitive function by examining how individuals with distinct language profiles perform on the NIH Toolbox Cognition Battery. In particular, the utility of employing a data-driven approach for identifying subgroups of participants was examined, which may provide a more nuanced and ecologically valid model of language experience than traditional monolingual-bilingual dichotomies. A cluster analysis based on self-reported ages of language acquisition and proficiency indicated that participants with second-language experience could be broadly grouped into “early” versus “late” bilinguals, as well as “balanced” versus “unbalanced” bilinguals. Early bilinguals acquired a second language at the same time as or soon after the first language (mean difference in ages of acquisition = 4.21 years), while late bilinguals acquired a second language substantially later than the first language (mean difference = 12.84 years). Balanced bilinguals had similar levels of first and second language proficiency (mean difference = 0.63 on a scale from 0 to10) and unbalanced bilinguals were relatively more dominant in their first than second language (mean difference = 6.63).

Overall, our results align with previous work indicating that greater bilingual language experience is associated with better performance on *executive control* tasks (e.g., Flanker task and DCCS test; see Bialystok, 2017 for a review), but weaker performance on tasks that rely on linguistic and verbal abilities within a single language (e.g., List Sorting Working Memory test; Bialystok et al. 2022; Gollan et al. 2005; Ivanova and Costa, 2008; Marian et al. 2018a). We found that balanced proficiency and early age of acquisition moderated performance on *executive function* tasks (i.e., DCCS test, Flanker task, and List Sorting Working Memory test), but not on *language-based* tasks (i.e., Picture Vocabulary test and Oral Reading Recognition test). Controlling for English proficiency, performance on picture vocabulary task (English vocabulary), picture sequence memory task (episodic memory), and pattern comparison task (processing speed) was comparable among the five cluster groups. Our findings indicate that the effects of language experience on cognitive function vary depending on both the *domain* of cognitive function (e.g., verbal vs. nonverbal) and the *dimension* of language experience (e.g., age of acquisition vs. proficiency).

The results from the flanker test indicate that inhibitory control may be jointly impacted by dual-language proficiency and age of acquisition. Flanker scores were significantly higher among early-balanced, late-balanced, and early-unbalanced bilinguals compared to late-unbalanced bilinguals. It has been hypothesized that effects of bilingualism on inhibitory control may stem from the parallel activation of both languages and the use of domain-general executive functions to minimize interference from the non-target language (Bialystok et al. 2012). Our finding that Flanker scores were highest among those with more balanced bilingual proficiency is consistent with prior work showing that high-proficiency languages are more likely to be activated when not in use (e.g., Blumenfeld and Marian, 2007; Canseco-Gonzalez et al. 2010; Meuter and Allport, 1999; Weber and Cutler, 2004), which necessitates a greater degree of inhibition to suppress competition. In addition, according to Cummins’ Threshold Hypothesis (Cummins, 1976), the benefits associated with bilingualism may only appear once the speaker has attained a certain level of competency in their second language. This could also serve as an explanation for why late-unbalanced bilinguals had lower scores on the executive function measures than the other bilingual groups. Balanced bilingualism may be especially important for measures of selective attention because early- and late-balanced bilinguals need to manage attention between two jointly activated languages. These findings highlight the need to examine how different forms of bilingual experience influence specific cognitive functions.

Effects of language experience on the Dimensional Change Card Sort (DCCS) test performance suggest that cognitive flexibility may be jointly impacted by age of dual-language acquisition and relative proficiency. DCCS scores were higher for early-unbalanced compared to late-unbalanced bilinguals. Additionally, DCCS scores were higher for early-balanced bilinguals than monolinguals and late-unbalanced bilinguals. The effects of bilingualism on cognitive flexibility have been attributed to bilinguals’ experience alternating between multiple linguistic systems, which may facilitate task-switching performance by enhancing global monitoring skills and maintenance of competing task goals (Barac and Bialystok, 2012; Soveri et al. 2011; Wiseheart et al. 2016). Early bilinguals have, by definition, a longer duration of experience managing multiple languages than late bilinguals. Hence, the advantages observed for cognitive flexibility may stem in part from a higher probability of switching between languages within a single context (e.g., with family). Indeed, although all subgroups in the current study reported greater L1 than L2 experience with family, the asymmetry in family language use was smaller among early bilinguals compared to late bilinguals (refer to Tables 2 and 3). Therefore, bilinguals with more experience using multiple languages for most of their lives showed greater cognitive flexibility than those with less experience using multiple languages.

On the executive control task that relied on verbal abilities, early-balanced bilinguals performed significantly worse than late-unbalanced bilinguals and marginally worse than monolinguals on the List Sorting Working Memory Test. These findings are likely driven by differences in English proficiency between groups. In our sample, the unbalanced bilinguals performed the NIH Toolbox Cognition Battery in their more dominant and proficient language (i.e., English). Late-unbalanced bilinguals (*M* = 9.73, *SD* = 0.51) rated their overall proficiency in English higher than early-balanced bilinguals (*M* = 9.16, *SD* = 0.99, *p* < 0.001). Prior work with children has shown that unbalanced bilinguals generally outperform balanced bilinguals on language-based tasks in their dominant language (e.g., Persici et al. 2019). The observed effects for English verbal working memory performance may stem from relatively less extensive English experience rather than effects on memory per se. Preliminary support for this interpretation comes from the finding that nonverbal picture sequence memory did not differ among the five cluster groups, suggesting that the lower list sorting scores among early-balanced bilinguals may be primarily attributed to demands associated with English language processing. Furthermore, this explanation is supported by a significant correlation between list working memory scores and average self-reported proficiency in English, *R* = 0.22, *t*(163) = 2.95, *p* = 0.0037, 95%CI [0.07 0.37]. Considering that bilinguals have difficulties in lexical access and retrieval compared to their monolingual counterparts (e.g., Gollan et al. 2005), early-balanced bilinguals may be performing less well than monolinguals and late-unbalanced bilinguals on the working memory task because it is verbal in nature (Calvo et al. 2016).

Future research should consider performing a cluster analysis with a) more than two dimensions (e.g., x-axis as age of acquisition, y-axis as proficiency, and z-axis as language usage or exposure), b) different measures of language experience and seeing which measures provide better clustering, or c) objective measures of language proficiency because self-assessments of proficiency can vary significantly between different bilingual populations (Tomoschuk et al. 2019). The inclusion of language usage and objective measures of language proficiency will allow for more fine-grained clustering and contribute to our understanding of how variability in bilingual language experience influences cognition.

Considering there is no standard definition for “bilingualism” and language background can be characterized by multiple dimensions (e.g., proficiency, age of acquisition, and so on; Byers-Heinlein et al. 2019), the current study used machine learning to examine the complexities and nuances associated with language experience. Specifically, we gained a better understanding of how variability in bilingual language experience shapes certain aspects of cognition and not others. Our results suggest that more balanced bilinguals may have enhanced inhibitory control abilities by virtue of having to select between two jointly activated languages. Furthermore, early bilinguals may have enhanced cognitive flexibility than late bilinguals due to their extensive experience in managing multiple languages at an early age.

In conclusion, the present study provides a model for how effects of language experience on cognitive function may be examined using a bottom-up and data-driven approach to identifying subgroups of participants. The interaction between first and second-language proficiency and age of acquisition has differential effects on nonverbal and verbal executive control, but no effect on processing speed and episodic memory among young adults. In addition to enabling more nuanced characterizations of linguistic profiles that extend beyond arbitrary dichotomies and isolated measures of cognitive function, comparisons of naturally occurring subgroups can provide a more holistic and ecologically valid perspective on how bilinguals differ among each other in and outside the lab. A multidimensional approach that considers variation in language experience and cognitive abilities as meaningful patterns in human behaviour can advance our understanding of how bilingualism shapes the mind and brain.

## Figures and Tables

**Table 1:** Summary of L1 and L2 measures for monolinguals and bilinguals.

	Measure	Group	L1	L2	L1 vs. L2

Proficiency (0–10)	Speaking	Monolingual	9.79 (0.49)	0.45 (1.5)	***
		Bilingual	9.36 (0.9)	6.02 (3.07)	***
	Reading	Monolingual	9.75 (0.65)	0.55 (1.88)	***
		Bilingual	9.17 (1.51)	6.33 (2.96)	***
	Understanding	Monolingual	9.79 (0.5)	0.55 (1.86)	***
		Bilingual	9.51 (0.78)	6.39 (3.03)	***
Age of Acquisition (in years)	Overall	Monolingual	0.36 (0.67)	19.76 (8.49)	***
		Bilingual	0.36 (0.83)	8.46 (4.82)	***
	Reading	Monolingual	3.86 (1.63)	18.96 (7.11)	***
		Bilingual	4.1 (1.89)	10.02 (4.24)	***
Manner of Acquisition (0–10)	Family	Monolingual	8.88 (1.65)	0.55 (2.13)	***
		Bilingual	9.27 (1.4)	2.75 (3.45)	***
	Friends	Monolingual	6.71 (2.6)	0.55 (2.08)	***
		Bilingual	6.9 (3.09)	6.77 (3.61)	
	Self-Instruction	Monolingual	1.48 (3.44)	0.03 (0.19)	
		Bilingual	1.79 (2.77)	3.4 (3.2)	***
	TV	Monolingual	5.46 (3.15)	0.28 (1.49)	***
		Bilingual	5.94 (2.89)	5.22 (3.12)	
	Radio/Music	Monolingual	3.67 (3.05)	0.17 (0.93)	***
		Bilingual	2.82 (2.74)	2.82 (3)	
	Reading	Monolingual	7.96 (1.97)	0.69 (2.58)	***
		Bilingual	7.3 (2.42)	7.21 (2.98)	
Current Exposure (0–10)	Family	Monolingual	9.62 (0.97)	0.59 (2.23)	***
		Bilingual	8.27 (2.54)	1.79 (2.36)	***
	Friends	Monolingual	9.75 (0.53)	0.21 (1.11)	***
		Bilingual	6.97 (3.26)	5.64 (3.87)	
	Self-Instruction	Monolingual	5.62 (4.73)	0 (0)	***
		Bilingual	2.05 (3.56)	1.88 (2.68)	
	TV	Monolingual	9.12 (2.17)	0.34 (1.86)	***
		Bilingual	6 (3.32)	4.42 (3.41)	**
	Radio/Music	Monolingual	9.04 (1.49)	0.24 (1.12)	***
		Bilingual	5.85 (3.18)	4.7 (3.36)	
	Reading	Monolingual	9.79 (0.51)	0.34 (1.86)	***
		Bilingual	5.5 (3.57)	6.25 (3.7)	
Immersion (in years)	Family	Monolingual	23.48 (4.31)	2.31 (6.93)	***
		Bilingual	20.49 (4.19)	3.76 (7.16)	***
	Country	Monolingual	23.72 (4.05)	0.83 (4.46)	***
		Bilingual	17.58 (7.13)	5.45 (7.22)	***
	School/Work	Monolingual	21.76 (5.13)	0.62 (3.34)	***
		Bilingual	16.08 (7.94)	5.65 (6.39)	***

*Note.* Monolinguals’ L2 ages of acquisition were set to their current ages and other experience measures were set to 0 when no second language was known. Values in parentheses represent standard deviations. Asterisks represent significant differences between L1 and L2.

*** *p* < 0.001

** *p* < 0.01.

**Table 2:** Composite L1 and L2 language experience measures by cluster group.

Measure	Late-Unbalanced	Early-Unbalanced	Late-Balanced	Early-Balanced

L1 AoA	0.06 (0.24)	0.39 (1.12)	0.14 (0.44)	0.67 (0.99)
L2 AoA	13.44 (1.76)	5.96 (3.04)	12.34 (3.17)	4.31 (2.33)
L1 Proficiency	9.73 (0.51)	9.90 (0.23)	9.47 (0.65)	8.81 (1.24)
L2 Proficiency	3.11 (1.20)	3.26 (1.33)	7.74 (1.31)	8.78 (1.27)
L1 Family Acquisition/Exposure	9.68 (0.93)	8.81 (1.44)	8.98 (1.35)	8.44 (1.89)
L2 Family Acquisition/Exposure	0.75 (1.27)	3.56 (3.53)	1.21 (1.95)	2.84 (2.85)
L1 Media/Community Acquisition	6.73 (2.05)	5.41 (2.14)	5.81 (1.76)	5.54 (1.78)
L2 Media/Community Acquisition	2.29 (1.43)	1.81 (1.00)	6.14 (1.52)	6.67 (1.65)
L1 Media/Community Exposure	8.98 (1.08)	8.78 (1.27)	5.47 (2.78)	5.10 (2.73)
L2 Media/Community Exposure	1.27 (1.00)	1.00 (0.89)	5.29 (2.61)	6.90 (2.27)
L1 Immersion	20.83 (2.45)	21.55 (3.73)	18.57 (7.13)	10.85 (7.56)
L2 Immersion	1.05 (2.44)	1.40 (2.68)	3.55 (3.98)	9.88 (7.06)

*Note.* Values in parentheses represent standard deviations.

**Table 3:** Composite L1 and L2 language experience measures by early versus late bilinguals and balanced versus unbalanced bilinguals.

Measure	Early Bilinguals	Late Bilinguals	Early vs. Late	Balanced Bilinguals	Unbalanced Bilinguals	Balanced vs. Unbalanced

L1 Age of Acquisition	0.58 (1.03)	0.10 (0.35)	***	0.48 (0.88)	0.19 (0.74)	*
L2 Age of Acquisition	4.80 (2.65)	12.94 (2.55)	***	7.11 (4.67)	10.42 (4.38)	***
L1 Proficiency	9.14 (1.16)	9.61 (0.59)	**	9.04 (1.11)	9.80 (0.43)	***
L2 Proficiency	7.13 (2.85)	5.24 (2.64)	**	8.42 (1.37)	3.17 (1.24)	***
L1 Family Acquisition/Exposure	8.49 (1.83)	9.26 (1.23)	*	8.60 (1.76)	9.36 (1.19)	
L2 Family Acquisition/Exposure	2.95 (2.93)	1.03 (1.71)	**	2.38 (2.72)	1.88 (2.74)	
L1 Media/Community Acquisition	5.51 (1.81)	6.19 (1.91)		5.62 (1.77)	6.25 (2.14)	
L2 Media/Community Acquisition	5.97 (2.32)	4.65 (2.40)	*	6.52 (1.62)	2.10 (1.27)	***
L1 Media/Community Exposure	5.63 (2.88)	6.90 (2.83)	*	5.21 (2.73)	8.90 (1.13)	***
L2 Media/Community Exposure	6.06 (2.97)	3.74 (2.90)	**	6.45 (2.46)	1.16 (0.94)	***
L1 Immersion	13.92 (8.25)	19.80 (5.23)	***	13.55 (8.25)	21.11 (3.00)	***
L2 Immersion	7.43 (7.22)	2.20 (3.44)	***	7.67 (6.84)	1.19 (2.51)	***

*Note.* Asterisks in the Early vs. Late column indicate significant differences between early bilinguals (early-unbalanced + early-balanced) and late bilinguals (late-unbalanced + late-balanced), while asterisks in the Balanced vs. Unbalanced column indicate significant differences between balanced bilinguals (early-balanced + late-balanced) and unbalanced bilinguals (early-unbalanced + late-unbalanced).

*** *p* < 0.001

** *p* < 0.01

* *p* < 0.05.

## Data Availability

The dataset analyzed for this study is available from the corresponding author upon reasonable request.
